# Impact of *rad*iographer *i*mmediate rep*o*rting of chest *X*-rays from general practice on the lung cancer pathway (radioX): study protocol for a randomised control trial

**DOI:** 10.1186/s13063-017-2268-x

**Published:** 2017-11-06

**Authors:** Nick Woznitza, Anand Devaraj, Samuel M. Janes, Stephen W. Duffy, Angshu Bhowmik, Susan Rowe, Keith Piper, Sue Maughn, David R. Baldwin

**Affiliations:** 1 Radiology Department, Homerton University Hospital, London, UK; 2 School of Allied Health Professions, Canterbury Christ Church University, Canterbury, UK; 3 Radiology Department, Royal Brompton and Harefield NHS Foundation Trust, London, UK; 4 Lungs for Living Research Centre, UCL Respiratory, University College London, London, UK; 5 Queen Mary University London, London, UK; 6 Department of Respiratory Medicine, Homerton University Hospital, London, UK; 7 City and Hackney Clinical Commissioning Group, London, UK; 8 Department of Respiratory Medicine, Nottingham University Hospitals, Nottingham, UK

## Abstract

**Background:**

Diagnostic capacity and suboptimal logistics are consistently identified as barriers to timely diagnosis of cancer, especially lung cancer. Immediate chest X-ray (CXR) reporting for patients referred from general practice is advocated in the National Optimal Lung Cancer Pathway to improve time to diagnosis of lung cancer and to reduce inappropriate urgent respiratory medicine referral for suspected cancer (2WW) referrals. The aim of radioX is to examine the impact of immediate reporting by radiographers of CXRs requested by general practice (GP) on lung cancer patient pathways.

**Methods:**

A two-way comparative study that will compare the time to diagnosis of lung cancer for patients. Internal comparison will be made between those who receive an immediate radiographer report of a GP CXR compared to standard radiographer GP CXR reporting over a 12-month period. External comparison will be made with a similar, neighbouring hospital trust that does not have radiographer CXR reporting. Primary outcome is the effect on the speed of the lung cancer pathway (diagnosis of cancer or discharge). Secondary outcomes include the effect of the pathway on efficiency including the number of repeat CXRs performed in a timely fashion for suspected infection and the effect of immediate reporting of GP CXRs on patient satisfaction.

**Discussion:**

The radioX trial will examine the hypothesis that immediate reporting of CXRs referred from GP reduces the time to diagnosis of lung cancer or discharge from the lung cancer pathway.

**Trial registration:**

International Standard Randomised Controlled Trial Number ISRCTN21818068. Registered on 20 June 2017.

**Electronic supplementary material:**

The online version of this article (doi:10.1186/s13063-017-2268-x) contains supplementary material, which is available to authorized users.

## Background

Lung cancer is the leading cause of cancer death worldwide [1]. When compared to other common cancers, the prognosis for lung cancer is worse [2]. In the United Kingdom (UK) there has been a recent modest increase in survival, with 12.6% of patients with lung cancer surviving for 5 years [3], although 30% of patients die within 90 days of diagnosis [4]. Diagnosis of lung cancer is often made at a late stage, when prognosis is poor [5], and several factors are thought to influence this. Symptoms suggesting lung cancer are often non-specific until late in the disease, which results in diagnostic difficulties in primary care [4, 6, 7]. In an attempt to address this, recent guidance by the National Institute for Health and Care Excellence (NICE) has lowered the threshold for investigation and referral to specialist care for cases of possible malignancy, including lung cancer (NG12) [8].

Imaging has become embedded into an increasing range of patient pathways, with the number of investigations performed in England doubling in 9 years [9]. Service challenges for radiology in the UK are threefold; sustained increases in activity [9, 10], a chronic shortage of consultant radiologists [11, 12] and unprecedented economic restrictions [13]. Recognising the need to improve patient outcomes for cancer, especially lung cancer which has shown minimal improvement in survival rates [2, 5], renewed focus is being given to rapid referral and diagnosis in cases of suspected cancer [6, 8, 14]. These initiatives will undoubtedly increase the volume of imaging investigations performed at a time when diagnostic capacity is failing to meet current demand [15].

A clinical report of imaging examinations is essential to guide diagnostic and treatment decisions. Time to a clinical report can be a serious factor in diagnostic delays [16–18] with recognition that small delays for lung cancer diagnosis may contribute to higher stage at diagnosis [19] and also a deterioration in performance status that may influence suitability for treatment. In the setting of the lung cancer pathway, delays are often multifactorial, but may be contributed to by the time taken to report a chest X-ray (CXR). This is because the very first step in the lung cancer pathway is often the identification and reporting of a lung mass on a CXR, which should then trigger a staging computed tomogram (CT). The use of appropriately trained radiographers to undertake clinical reporting is not new. Skeletal radiograph reporting, for example, has become widespread across the UK [12, 20], and in many departments provides a significant contribution to reporting capacity [21, 22]. More recently, reporting radiographers have been trained to report CXRs [23, 24] and this has been proposed as a method of minimising CXR reporting times in patients with suspected lung cancer [25]. There is some limited evidence to date that has evaluated CXR accuracy rates of trained reporting radiographers in comparison with radiologists. Reporting radiographers (*n* = 40) were found to have high sensitivity (95.4%; 95% CI 94.4–96.3%) and specificity (95.9%; 95% CI 94.9–96.7%) at an objective, structured examination of 100 CXRs at the completion of an accredited training programme [23].

Recent work found poor compliance with suggested optimal diagnostic investigations for lung cancer, with 23% of patients in England receiving investigation and results within the recommended timeframes with significant variation between regions [26]. This study aims to evaluate the impact of radiographer reporting on the timeliness, accuracy and quality of CXR reports, as well as the impact on the overall lung cancer pathway in comparison with radiologists. These parameters have not previously been studied in lung cancer patients. The current study could act as a pilot study for a larger, multisite evaluation if results are positive.

## Methods

The aim of the current study is to investigate the impact of radiographer immediate CXR reporting on the lung cancer pathway.

### Trial design

**Fig. 1:**
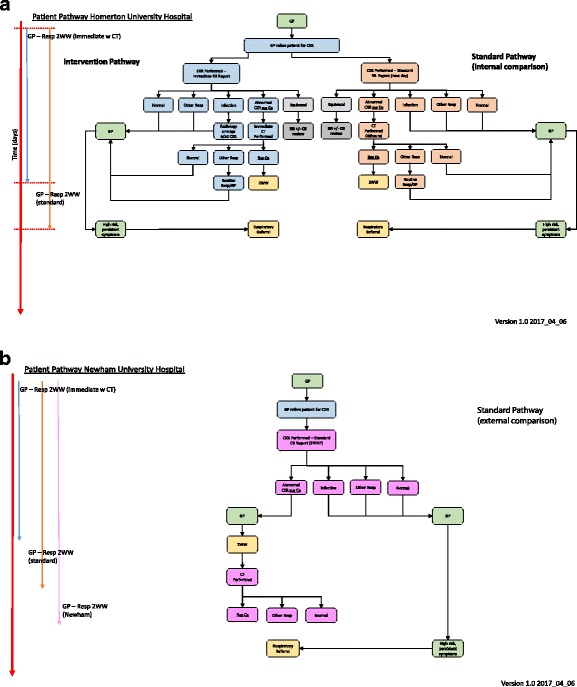
Intervention and standard patient pathway at Homerton University Hospital and Newham General Hospital (external comparator). *GP* general practitioner, *CXR* chest X-ray, *CT* computed tomography, *RR* reporting radiographer, *CR* consultant radiologist, *Other Resp* other respiratory disease, *sus CA* suspicious for cancer, *2WW* urgent respiratory referral for suspected cancer, *Routine Resp* routine referral to respirator medicine

**Fig. 2:**
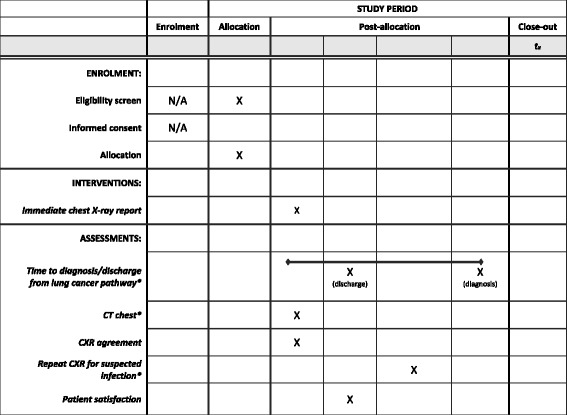
Schedule of enrolment, interventions and assessments. C*XR* chest X-ray, *CT* computed tomography scan, *when required

A two-way comparative study that will compare the time to diagnosis of lung cancer for patients. Internal comparison will be made between those who receive an immediate radiographer report of a GP CXR compared to standard radiographer GP CXR reporting (Fig. 1). The intervention group will receive an immediate CXR report and be offered a CT for CXRs suspicious for cancer. The control group will have the CXR reported no later than next working day in line with current protocols. Key protocol elements are summarised in the SPIRIT (Standard Protocol Items: Recommendations for Interventional trials) 2013 Checklist [27] (Additional File 1) and Figure (Fig. 2).

The diagnostic aspect of the lung cancer pathway at Homerton University Hospital is relatively streamlined. To enable comparison with radiology service delivery at other institutions’ time to diagnosis (immediate and standard CXR reporting) will be compared with Newham University Hospital (Fig. 2). This adjacent hospital has comparable patient demographics, a similar number of lung cancer patients per year and is of comparable size. Newham does not currently have CXR-reporting radiographers and does not offer a straight-to-CT service for CXRs suspicious for lung cancer.

### Study setting

Research Ethics Committee and Health Research Authority approval was granted on 6 June 2017 (REC 17/LO/0870; HRA 221968). This study will not directly recruit patients; it is an evaluation of health service delivery and, as such, no patient consent is required. Intervention is at an institutional level and institutional approval has been gained. No additional or different tests will be performed, and all the reporting practitioners (reporting radiographers and consultant radiologists) currently report CXRs in clinical practice. The comparative aspect of the study is the timing, accuracy and usefulness of the CXR report; immediate compared to standard care. Patient-identifiable data will not be available outside of the direct clinical care team, only anonymised data will be used. Patients will be assigned a unique study identifier at time of CXR by the clinical care team. Block randomisation, institutional rather than patient enrolment and the use of de-identified data is in line with previous research that has examined the order of interpretation between readers [28]. The intervention is considered to be an alternative, non-inferior form of standard practice since radiographer reporting of CXRs has already been implemented in some NHS trusts in the UK. Radiographer reporting, including CXRs, has been shown to create additional diagnostic capacity at centres that have embedded this into the imaging department [21, 22, 29]. However, the published evidence on radiographer reporting of CXRs is limited. Furthermore, robust methods of evaluating diagnostic reports (including actionability and usefulness) of radiographers and radiologists using independent experts has not previously been attempted.

Clinical assessment will be made by a general practitioner and a referral made to Homerton University Hospital for a CXR examination following standard and established referral procedures. The referral for CXR will be checked by the performing radiographer or supervised assistant practitioner to ensure that the referral meets Ionising Radiation (Medical Exposure) Regulations (IRMER) (2000) requirements and adheres to departmental protocols for a justified referral. Chest X-rays will be obtained using digital radiography equipment, and radiation doses will be as low as possible while maintaining good image quality. Existing departmental imaging protocols will be followed. The standard X-ray projection for a chest examination is a single posterior-anterior (PA) X-ray. The radiographer or assistant practitioner will check all images for diagnostic quality and record the radiation dose on the Radiology Information System (RIS) in line with departmental standard operating procedures. If the radiographer or assistant practitioner performing the CXR identifies a potentially significant abnormality; for example, lung cancer or pneumothorax, this will be triaged for an immediate report according to current protocol.

Inclusion and exclusion criteria are presented in Table 1.

**Table 1:** Inclusion and exclusion criteria

	Criteria
Inclusion	• Referred for a chest X-ray from general practice
	• Aged over 16 years
Exclusion	• Active diagnosis of lung cancer

### Randomisation

Intervention is at an institutional level; individual patients will not be randomised. Half-day sessions will be randomised to intervention or standard practice, using a randomisation list provided by the study statistician. This is in line with previous studies that have examined the timing or order of X-ray reading but where all examinations are requested as part of routine clinical care and receive reports from the same practitioners [30].

### Intervention

The intervention reporting strategy is modelled on the National Optimal lung cancer pathway developed in 2016 [31]. The intervention strategy aims to streamline the patient journey through the lung cancer pathway by providing prompt interpretation of CXRs referred by general practice (GP) and offering immediate CT when appropriate.

Chest X-rays included in the intervention arm will be reported at the time of image acquisition while the patient is still in the radiology department. Patients who have a CXR suspicious for cancer will be offered an immediate CT of the chest and upper abdomen.

### Control

Current practice in most radiology departments is for GP examinations to be reported once the patient has left the department. Considerable variability exists across England in the time taken to report X-ray examinations (report turnaround time; RTAT). At Homerton University Hospital, all GP X-rays are reported during the next reporting session following examination, with a maximum RTAT of one working day. Patients who have a CXR suspicious for cancer are offered an appointment for a CT of the chest and upper abdomen via the radiology department secretary team, with the results sent to the referring GP and the cancer referrals office. Current practice is that if a suspected abnormality is identified by the radiographer who performs the CXR an urgent report (reporting radiographer or consultant radiologist) is arranged while the patient is still in the department. If the findings are suspicious for lung cancer the patent is offered a CT of the chest and abdomen. This protocol will continue throughout the study for the control reporting sessions.

### Outcome measures

The primary outcome is to test the impact of radiographer immediate reporting of GP CXRs, with immediate CT where appropriate, on time from performance of the CXR to treatment (with intermediate time points)/discharge for lung cancer.

Secondary outcome measures include:

- Measurement of the effect on the speed of the lung cancer pathway:
  (i) 6-and 12-month survival (lung cancer and all-cause)
  (ii) Number of emergency admissions for lung cancer
  (iii) Performance status at time of decision to treat
  (iv) Stage at diagnosis of lung cancer
- Measurement of the effect of the pathway on efficiency including:
  (i) The impact of immediate GP CXR reporting on the number of urgent respiratory cancer (2WW) referrals
  (ii) The accuracy and usefulness of radiographer CXR reporting in clinical practice
  (iii) The cost-effectiveness of radiographer reporting
  (iv) The influence of immediate GP CXR reporting, with immediate CT where appropriate, on the number of first 2WW appointments with all radiology results available
- Measurement of the number of repeat CXRs performed in a timely fashion for suspected infection
- The effect of immediate reporting of GP CXRs on patient satisfaction

In addition to comparison as per randomisation within Homerton University Hospital, primary outcomes will be compared with a neighbouring hospital, Newham University Hospital.

## Components of the chest X-ray reporting pathway

### Reporting radiographer chest X-ray report

All reporting radiographers participating in the study have completed an accredited postgraduate certificate in adult CXR reporting (experience 1–8 years) and currently provide CXR reports in clinical practice. All CXRs referred by GP on eligible patients (aged over 16 years, no active history of lung cancer) will receive a reporting radiographer report. In line with current practice, a narrated report will be provided rather than a structured report. Image interpretation will occur on Picture Archiving and Communication System (PACS) workstations and the report entered into PACS and transferred to the patient electronic record. If the reporting radiographer requires additional investigations (repeat X-ray due to inadequate initial X-ray, additional X-ray view, CT of the chest and abdomen), these will be arranged by the reporting radiographer at time of the CXR report.

### Off-protocol radiographer reporting

Where the radiographer performing the CXR is concerned about the appearance of the X-ray or by the clinical condition of the patient, current practice at Homerton University Hospital is for the CXR to be reviewed by a reporting radiographer or a consultant radiologist prior to the patient leaving the department. This includes, for example, where the radiographer suspects a pneumothorax, tuberculosis or cancer. If a radiographer has concerns that the appearances of the CXR is abnormal and a significant pathology may be present, these patients will receive an immediate report, regardless of the reporting session allocation (immediate/standard) so as not to negatively impact on patient management. All such occurrences will be identified, included in the intention-to-treat principle, but we will also carry out sensitivity analysis excluding them. In view of randomisation, we expect the same rates of such cases in intervention and control sessions.

### Equivocal reporting radiographer reports

For cases where the reporting radiographer is unsure with the findings, and/or clinical significance of the CXR, they will be free to review the case with another reporting radiographer and/or consultant radiologist. This is in line with current best practice. This will include, for example, instances where previous cross-sectional imaging is available for the patient, or where there may be unfamiliar medical terminology on the CXR request form. All occurrences will be recorded.

### Consultant radiologist chest X-ray report

All CXRs will receive a consultant radiologist report (general radiologists; experience range 2–21 years post FRCR), blinded to the reporting radiographer CXR report. Consultant radiologist reporting will occur at the next session following the reporting radiographer report. Interpretation will occur using PACS workstations and the report will be entered into a secure database.

### Comparison of radiographer and radiologist reports

The CXR reports generated by the reporting radiographers and consultant radiologists will be extracted, anonymised for source of report (radiographer/radiologist) and entered into a secure database using the unique study identifier. A respiratory physician will compare the reports for discrepancies, using a proforma with predefined criteria for clinically significant abnormalities. Discrepancies in observations, interpretations and recommendations will be highlighted. These criteria have been previously validated [32]. Report comparison will occur within three working days of the CXR examination.

### Additional radiology investigations

All additional radiology investigations will be organised by the radiology department following established departmental operating procedures. These additional investigations would be performed as part of routine clinical practice and will not require any additional radiation exposure. The reporting radiographers, after appropriate training, have been designated ‘non-medical referrers’ according to IRMER 2000 legislation.

### Repeat chest X-ray for suspected infection

According to British Thoracic Society (BTS) guidance [33], patients who have a CXR that is suspicious for infection require a follow-up CXR 6 weeks later following antibiotic therapy to ensure resolution. The reporting radiographer will arrange the follow-up CXR at the time of the initial CXR report for the immediate reporting arm, and the patient will be asked to re-attend the radiology department in 6 weeks. This will be communicated in the CXR report.

For patients who have a CXR suspicious for infection in the standard care arm the recommendation for a follow-up CXR in 6 weeks will be included in the report conclusion. This will be requested by the general practitioner, as is current practice.

### CT of the chest

Patients who have an abnormal CXR suspicious for cancer will have a CT of the chest performed. The reporting practitioner (reporting radiographer or consultant radiologist) will arrange this following standard department procedure. The CT scan forms part of routine clinical management and, therefore, does not require any additional radiation exposure. A consultant radiologist will interpret all CTs.

The CT performed will be stratified based on the CXR appearances and the likelihood of cancer. This will minimise radiation exposure, in line with best practice. For patients with a CXR that is suspicious but not categorical for lung cancer, a low-dose, unenhanced CT of the chest will be performed. For patients who have a CXR that shows a high likelihood of cancer, a CT of the chest and abdomen with intravenously administered contrast will be offered

### Index diagnosis by thoracic radiologist

Chest X-rays that are found to have discordant reporting radiographer and consultant radiologist reports at peer review will have an index diagnosis. For cases that have undergone a subsequent CT scan of the chest and abdomen, the CT report will constitute the index diagnosis. CXR reports, either reporting radiographer or consultant radiologist, will be deemed a true positive if CT confirms the CXR diagnosis and a false positive if the CT is normal or another pathology is demonstrated. True positive and true negative will be a consensus decision and corroboration between the CT and clinical history between a respiratory physician and a thoracic radiologist. Assessment of report accuracy will be made blinded to the origin (reporting radiographer/consultant radiologist) of the CXR report.

For cases that have not had a CT performed, an independent expert thoracic consultant radiologist will constitute the index diagnosis. The index radiologist will feed back the diagnosis via a standardised proforma. All available thoracic imaging (X-ray, CT) for the patient will be sent via the Image Exchange Portal (IEP) to the Royal Brompton Hospital. IEP is an established, secure method of transferring radiology cases for external review within the NHS. A thoracic consultant radiologist will review the available imaging and provide the definite diagnosis. CXR reports, both reporting radiographer and consultant radiologist, will be deemed a true positive if the thoracic radiologist confirms the CXR diagnosis and a false positive if the thoracic radiologist interpretation is normal or another pathology is demonstrated.

### Statistical considerations

#### Sample size

For the primary endpoint in this pilot study, time to treatment decision for lung cancers, if we expect an 11-day advance in time to first treatment decision, with a standard deviation of 14 (previous audit data suggest this degree of variation), 26 cancers in each group will confer 80% power (two-sided testing, 5% significance level), for the internal randomised comparison. We expect around 50 cancers per year in Homerton University Hospital (HUH), so we will have adequate power for this difference. A reduction in time to diagnosis of 2 weeks was found to improve mortality of lung cancer patients so this difference could be clinically significant in the current pilot study [34]. If we anticipate a 12-day, instead of an 11-day advance in diagnosis, we would only need 22 in each arm, 44 cancers in all, for 80% power. For the external comparison, assuming that Newham University Hospital has a similar number of lung cancers per year, therefore, we would have close to 90% power for the same difference and standard deviation. If we also compare times to diagnosis for all persons referred to the pathway (lung cancer and non-lung cancer diagnoses), previous data suggest an average of 18 days and a standard deviation of 14. If the intervention improves this by 7 days on average, with a standard deviation of 15, we would need 73 subjects in each group referred to the pathway to achieve 80% power (two-sided testing, 5% significance level). Thus, both the internal and external comparisons will be adequately powered.

#### Data analysis

Times to diagnosis, treatment and other continuous outcomes will be compared using simple *t* tests. Categorical outcomes, such as proportions of emergency admissions, will be compared using Poisson regression. Survival will be compared using proportional hazards regression. Patient satisfaction will be recorded in categorical outcomes, and will be compared using non-parametric tests.

#### Patient satisfaction

Patients referred for a CXR from GP will be identified by the radiology administration team, as is current practice. Eligible patients will have a Patient Satisfaction Survey posted to their home address, with a stamped self-addressed return envelope. No patient-identifiable data will be collected. Comparison will be made between patients who received an immediate and routine CXR report. The Patient Satisfaction Survey to be used has been included as an Appendix.

#### Health economic assessment

Adaptation of a health economic model that examined the impact of radiographer CXR reporting on the lung cancer pathway will be performed [35]. The model for this project will map out the care pathways following standard reporting and immediate reporting. It is assumed that differences in time to treatment will affect severity and, hence, costs and quality of life. Costs will be calculated from an NHS perspective, covering a 1-year period, and include X-ray reporting time, CXR cancer and non-cancer diagnostic accuracy, subsequent care costs, as well as reading and supervision costs. The cost per case detected will be reported. Quality-of-life scores will be obtained from the academic literature for different cancer stages and these will be used to generate quality-adjusted life years (QALYs). One-way and probabilistic sensitivity analyses will be conducted to assess the impact on costs and cost-effectiveness of changing parameters in the model. Due to the timing of the intervention in relation to the lung cancer pathway there may be no meaningful difference in QALYs for the internal comparison. The reduction in time to a non-lung cancer diagnosis may be a worthwhile improvement in quality of life.

## Discussion

The current study will determine the effect of immediate reporting of CXRs referred from GP, with immediate CT where appropriate, on the time to diagnosis of lung cancer. Although only one part of the patient pathway, immediate GP CXR reporting could positively impact lung cancer diagnosis and outcomes in at least three ways: firstly, by providing an immediate CXR report and initiating earlier further investigation including CT, the time to diagnosis will be shortened. There is debate within the academic literature as to the significance of this in terms of improvements in early survival times, performance status and reducing emergency admissions [34]. The current study will examine this, both with internal and external comparison. Secondly, the efficiency of the service may be improved by reducing the number of lung cancer pathway referrals through early provision of an alternative diagnosis, which in turn means less time for patient anxiety and distress. Thirdly, the proposal may release consultant radiologist time that can instead be used to interpret more complex cross-sectional imaging and support interventional procedures including lung biopsy. A reduction in average time to diagnosis for lung cancer will help centres meet the ambitious target of 90% of lung cancer patients definitively diagnosed within 28 days by 2020 [14].

Diagnostic capacity is a significant barrier to improved outcomes for cancer patients [14, 36], with prompt radiology reports a particular issue across England [15, 18].

The limitations of the current study include the fact that the intervention occurs only at a single clinical site at which the diagnostic aspect of the lung cancer pathway is already relatively streamlined. This is addressed by external comparison with a neighbouring hospital with similar patient characteristics and a comparable number of lung cancers diagnosed annually.

## Trial status

Study protocol version 1.5 of 2 May 2017. The study will commence on 1 July 2017 and close on 30 June 2018. The trial was registered (ISRCTN21818068) on 20 June 2017.

## Appendix

### Patient Satisfaction Survey

Homerton University Hospital strives to offer effective, patient-focused healthcare. In order to improve services we would value your feedback on your experiences when you recently attended the radiology department for a chest X-ray. Please indicate your response to each question by circling the appropriate answer.

All answers are anonymous and confidential. If you have any questions please contact Dr. Nick Woznitza, radiographer, on 0208 510 7848.

Please return the completed survey in the stamped, self-addressed envelope provided.

**Q1 What is your gender?**

Male

Female

Prefer not to answer

**Q2 Which age group do you belong to?**

16–24

25–34

35–44

45–54

55–64

65–74

75–84

85 +

**Q3 To which of these ethnic groups do you consider you belong?**

White

1. English/Welsh/Scottish/Northern Irish/British

2. Irish

3. Gypsy or Irish Traveller

4. Any other White background, please describe

Mixed/Multiple ethnic groups

5. White and Black Caribbean

6. White and Black African

7. White and Asian

8. Any other Mixed/Multiple ethnic background, please describe

Asian/Asian British

9. Indian

10. Pakistani

11. Bangladeshi

12. Chinese

13. Any other Asian background, please describe

Black/African/Caribbean/Black British

14. African

15. Caribbean

16. Any other Black/African/Caribbean background, please describe

Other ethnic group

17. Arab

18. Any other ethnic group, please describe

Prefer not to answer

**Q4 When were you told that the results of your chest X-ray would be available?**

Immediately – given by a radiographer

Immediately – to contact my GP

Next day – to contact my GP

**Q5 Did you require any further tests?**

Yes – done at the same time as the chest X-ray

Yes – done on another day after the chest X-ray

No

**Q6 How do you feel about how you were told that you needed further tests?**

I did not need any further tests

It was done sensitively

It could have been done a bit more sensitively

It could have been done a lot more sensitively

**Q7 How did you feel about needing further tests**?

Frightened

Angry

Upset

Pleased that something was happening

Prefer not to say

Any comments?

**Q8 Would you have liked to be contacted by your own GP (Doctor) before the CT scan – even if this meant a delay to your scan?**

Yes

No

Not sure

Prefer not to say

**Q9 How long did you wait for your results after you had your CT scan?**

Less than a week

1–2 weeks

More than 2 weeks

Can’t remember

**Q10 If you had an appointment, was the booking system flexible enough for you?**

My scan was performed immediately

Yes

No

Don’t know/Can’t remember

**Q11 If you have any suggestions or comments about the service you would like to make, please use the space below**

## Additional file

Additional file 1: SPIRIT 2013 Checklist: recommended items to address in a clinical trial protocol and related documents*. (DOC 120 kb)

## Abbreviations

2WW: Urgent respiratory medicine referral for suspected cancer
BTS: British Thoracic Society
CR: Consultant Radiologist
CT: Computed tomography
CXR: Chest X-ray
GP: General practice
IEP: Image Exchange Portal
IRMER 2000: Ionising Radiation (Medical Exposure) Regulations
NHS: National Health Service
NICE: National Institute for Health and Care Excellence
PACS: Picture Archive and Communication System
PA: Posterior-anterior
QALY: Quality-adjusted life year
RIS: Radiology Information System
RR: Reporting Radiographer
RTAT: Report turnaround time
